# Anabolic-androgenic steroid users receiving health-related information; health problems, motivations to quit and treatment desires

**DOI:** 10.1186/s13011-019-0206-5

**Published:** 2019-05-16

**Authors:** Ingrid Amalia Havnes, Marie Lindvik Jørstad, Christine Wisløff

**Affiliations:** National Advisory Unit on Substance Use Disorder Treatment, Division of Mental Health and Addiction, Oslo University Hospital, PO Box 4959, Nydalen, 0424 Oslo, Norway

**Keywords:** Anabolic androgenic steroids, Performance-enhancing drugs, Doping, Adverse effects, Physical health, Mental health, Substance use, Addiction treatment, Substance use disorder treatment, Health service, Qualitative

## Abstract

**Background:**

Anabolic-androgenic steroids (AAS) are used to increase muscle strength and improve appearance, but users also carry the risk of developing physical and mental health problems. In Norway, the substance use disorder treatment system provides health care to this patient group, but few AAS users have sought such treatment. Therefore, a service was created to inform AAS users and next of kin of potential negative consequences and their treatment options. This study describes health problems, motivations for AAS cessation, and treatment desires among AAS users.

**Methods:**

Over four years, 232 AAS users and 60 next of kin contacted the information service and received an hour-long information session with healthcare personnel. Information about AAS use, physical and mental health problems, substance use, motivation for cessation, and whether the information seeker desired treatment were registered. Qualitative interviews were conducted among seven individuals and analyzed thematically to explore information service experiences.

**Results:**

Of the 232 AAS users, 179 (77.2%) desired treatment after completing the information session and 53 (22.9%) were unsure or did not want treatment. Those who desired treatment were significantly older, had used AAS longer, reported more physical and mental health side effects, and a higher proportion reported having children than those who did not desire treatment. Although 181 (78.0%) reported co-occuring physical and mental health problems, mental health problems were the most common motivation for AAS cessation (*n* = 108, 47.8%), followed by a combination of mental and physical health problems (52, 23.0%). Findings from qualitative interviews suggest that barriers to treatment may be overcome with an easily accessible service that informs about addiction treatment and facilitates the treatment entry process.

**Conclusions:**

Healthcare professionals who encounter users of AAS should have knowledge about AAS use and adverse effects. The desire for health care reveals extensive health problems and the user group is so non-homogeneous that examination and treatment must be adapted individually with focus on physical, mental and social factors as well as possible dependence of AAS and/or psychoactive substances.

## Background

Anabolic-androgenic steroids (AAS) include male sex hormones such as testosterone and synthetic derivatives with similar structure and effect [1]. AAS was mostly used by professional athletes until the early 1980s when recreational athletes began using AAS to increase muscle strength and to improve appearance and performance [2–4]. Estimates of the lifetime prevalence rates of AAS use in general populations varies, but is found to be higher in the United States [5], parts of Europe and the Middle East and lower in other parts of Asia and Africa [4, 6, 7]. Subpopulations such as former power elite athletes [8, 9], recreational athletes [4], injecting drug users [10, 11] and arrestees/prisoners [12, 13] report higher lifetime AAS use than the general population. In Norway, life time AAS use is estimated to be about 2–3% among men and less than 1% among women [14] and current AAS use is found to be 0.6% in an online survey of self-selected participants [15].

AAS used in supra-physiological doses have a diminishing effect on the hypothalamus pituitary gonad axis and may result in reduced endogenous production of testosterone. After termination of AAS use, temporary or lasting hypogonadism with symptoms such as depression, fatigue and sexual dysfunction may occur [16, 17]. AAS is often used cyclically with breaks between [18] to restore the body’s own production of testosterone. Between 20 and 50% of users seem to develop a form of AAS dependence [19–22]. Dependent users often use higher doses and take shorter breaks than planned before the cycle, or use AAS continuously, despite adverse effects. Mechanisms behind this addiction may be body image disorder, activation of the reward system during use, and/or mental and physical health problems as symptoms of hypogonadism after discontinuation [21–23].

The use of AAS is associated with a wide range of physical side effects, such as hematological, metabolic and cardiovascular diseases [2, 3, 24–27], anatomical changes in the brain [19], reduced cognitive function [28, 29], hepatic impairment, and disturbance of the sexual hormone system [17]. Men can develop gynecomastia and women can experience increased masculinization [2]. In addition, sexual function may be affected, in which increased libido during AAS use is often followed by decreased libido and erectile dysfunction upon discontinuation [16, 17, 30]. Some users may develop hypomania, manic or psychotic symptoms during AAS exposure, while post-cessation periods may be accompanied by depressive symptoms, anxiety and sleep disorders [2, 31–33]. AAS use is also found to be associated with aggressive behavior [31, 34–36], especially when combined with psychoactive substances [37]. Substance use, such as cocaine, amphetamines, cannabis and benzodiazepines, and use of hormones and other substances to reduce the side effects, is frequently reported by AAS-users [2, 38–43].

Although there is expected to be a rising number of AAS users with health problems due to longtime use, few AAS users seek treatment [3, 44]. Importantly, AAS users experience highly desirable effects during AAS use and may seek treatment only when the negative effects outweigh the desired effects [45].

In 2012, the specialized treatment system for substance abuse disorders (SUD) in Norway was given responsibility for providing health care to AAS users in need of treatment, and their next of kin. Use and possession of AAS and other doping agents became illegal in 2013, when the Norwegian Drug Act was amended. The National Steroid Project was established in 2014 at Oslo University Hospital to train health professionals in treating AAS-related health problems, and to inform users and their next of kin, as well as the general public, about health consequences related to AAS use and treatment options. Few AAS users have applied for or received treatment within the Norwegian SUD treatment system. Therefore, the Steroid Project created a free and voluntary information service, where users and their next of kin can contact health personnel within the specialist health care system directly with no need for referral, to receive information about AAS-related health problems and their treatment options in primary and specialist health care. This study has been conducted to describe the individuals seeking health-related information, the AAS users’ perceived AAS-related health problems and motivations for AAS cessation, to compare characteristics of the group of AAS users who desired treatment with the group of AAS users that were unsure or did not want treatment and to describe experiences of the information service.

## Method

This is a cross-sectional prospective study collecting data from 292 information sessions with AAS users and next of kin over a period of 4 years from 2015 to 2019. Additionally, a subset of data from a qualitative study exploring barriers to seeking treatment among AAS users have been included to provide examples of information session experiences.

### Setting

Individuals with SUD have treatment rights as patients in the Norwegian SUD treatment, which is publicly funded and widely available [46]. In 2016, 33,000 individuals received SUD treatment in Norway, and three out of four were in outpatient treatment [47]. In Norway, individuals with AAS-related health problems can seek primary health care mainly provided by general practitioners who together with the patient will decide whether a specialist referral to somatic specialized health care is necessary. Those who struggle to stop their AAS use and/or have psychosocial health problems related to former or present AAS use can be referred to outpatient SUD treatment [48]. The treatment goal in Norwegian SUD treatment for AAS users is developed together with the patient, and may be to end AAS use and/or reduce the health consequences related to use. Furthermore, in the Norwegian SUD treatment program, next of kin such as partners, parents, other relatives or close ones, may have their own treatment rights aimed to reduce their health and social costs.

### The information service

The national information service is aimed at AAS users with health problems and their next of kind in all health regions in Norway. The service is free, voluntary and anonymous and the information seekers contact the service directly to have a personal or telephone meeting with a health professional within the specialist health service without referral.

The session highlighted physical and mental side effects, social ramifications, illicit substance use in combination with AAS and legislation. Examination and treatment in primary health care, within the substance use disorder treatment system and other specialist health services, what kind of treatment results one can expect and information about the referral process were also covered in these meetings. The service users could also ask questions.

The service was announced on various internet sites for health services, through articles in national newspapers, letters to GPs, Anti-doping Norway, flyers delivered to somatic hospitals and during lectures for mainly health workers, but also for the police and prison authorities. In April 2018, the Steroid project at the Norwegian National Advisory Unit on SUD treatment launched a campaign on social media for the general public, AAS users and their next of kin. The campaign redirected the interested internet users to a web page with information about AAS, wanted and unwanted effects, treatment possibilities, and the information service [49].

### Data collection

This study included *all* users of AAS and next of kin who voluntarily contacted the Steroid Project for a health-related information session, over a period of about 4 years from April 2015 to March 2019. The information sessions were conducted by telephone or personal meetings lasting approximately one hour, and two via e-mail. Information seekers who contacted the service more than once were only registered the first time and all information seekers had individual information sessions. The AAS users reported health problems they perceived to be related to AAS use and the next of kin reported observed mental health and behavioral change after their relative/partner started to use AAS. Gender, age, occupation, marital status, number of children, age at first time use of AAS, number of years of AAS use, and combined use of illicit and/or addictive substances together with AAS were recorded.

The service provider recorded one or several of the following motivations to cease AAS usage: physical and/or mental health problems and other motivations. After the sessions, the service provider registered how the information seeker became aware of the service, whether the AAS user or their next of kin wanted a referral to the SUD treatment system or if they wanted treatment in primary health care or other parts of the specialist health care system after the information session. They were also asked whether they experienced the information service as useful to be able to decide whether they wanted SUD treatment.

### Cathegorization

All health problems and behavioral change were recorded during and after the information session as described by the information seeker and categorized by the three authors (MD/psychiatrist, Mpsych, registered nurse). The first categorization was conducted after the first 100 information sessions and some new categories were developed subsequently for side effects that had not been recorded previously.

*Mental health problems* were categorized as anxiety, depression, AAS dependence, sleep problems, experience of reduced empathy, anger/aggression, jealousy/paranoia, and behavioral change. Behavioral change included the following: social isolation, focus on food and exercise, megarexia, changed environment, reduction in ability to work, reduced or increased activity level, self-centeredness, loss of accountability, feelings of shame/embarrassment, low self-esteem, stronger emotions and experience of personality change. The reported *physical side effects* were categorized as appearance (acne, oily skin, thin skin, gynecomastia, striae, increased body hair, beard growth, hair loss, masculine appearance), sexual function / fertility (impotence, reduced fertility, infertility, reduced and increased libido), cardiovascular disease (cardiac hypertrophy, arrhythmia, tachycardia, myocardial infarction, hyperlipidemia, atherosclerosis, cerebral infarction, blood clot, polycythemia), muscle/skeletal disorders (muscle and tendon damage, pain conditions, muscle cramps, abscesses), endocrine disorders (hypogonadism, absent menstruation, deepened voice, testicular atrophy), and other (headache, sweating, memory impairment, frequent urination, infections, bloatedness, gastrointestinal symptoms).

### Analysis

Data was processed and analyzed using SPSS, version 25, and presented descriptively with simple statistics including measures of central tendency (mean, median), measures of dispersion (range, standard deviation). There were missing data for several variables, but the percentages displayed in the results section are based on total number of participants. To detect the differences in background variables between those who desired treatment with those who did not desire treatment, independent-samples *t*-tests were used. Differences in proportions were evaluated by Chisquare test. Complete cases were analyzed (Table 1). *P*-values < 0.05 were considered statistically significant.

**Table 1:** Description of background variables and mean number of side effects for information seekers who reported AAS use (*n* = 232)

	Treatment desire (*n* = 179)			No treatment desire/maybe (*n* = 53)				
	M	SD	Missing	M	SD	Missing	t	P
Age (years)	32.5	11.6		27.8	9.0		3.15	.002
Age AAS debut (years)	21.4	6.3	2	20.1	3.4	3	1.93	.056
Length AAS use (years)	10.9	8.3	2	7.6	7.2	3	2.57	.011
Physical health problems	1.7	1.1		1.2	.8		3.63	.000
Mental health problems	2.7	1.2		1.5	1.2		6.11	.000
Total health problems	4.4	1.7		2.7	1.4		6.50	.000
	n (%)			n (%)				
Partner	92 (57.5)		19	15 (42.9)		18		.115
Have children	68 (45.0)		28	7 (18.4)		15		.003
Report substance use	92 (80.0)		64	18 (78.3)		30		.850

### Qualitative data - information service experiences

To explore information service experiences, a subset of data was drawn from an ongoing qualitative study about barriers to seeking treatment for AAS users. Inclusion criteria were that participants had used AAS and experienced health problems with or without seeking health services and 21 participants were included altogether. The interview guide was developed together with a panel of five former AAS users and covered; positive and negative experiences of AAS use, health problems with or without treatment experiences, understandings of reasons not to seek help, methods to avoid or handle side effects, treatment needs, views on health services, and understandings of legal matters related to AAS use. The semi-structured interviews lasted about an hour, were audio recorded, transcribed verbatim and analyzed thematically [50, 51]. A researcher (TSS) conducted the majority of the interviews and the initial coding. Two of the authors (IAH, MLJ) reviewed the initial coding and searched for themes in joint discussions with TSS, to reach consensus. The subset of data concerning experiences with and/or understandings of the information session underwent the final two steps of thematic analysis (reviewing themes and producing the report) by IAH to be included in this article. To avoid researcher bias, the following actions were taken; a) the interview guide was developed with former AAS users, b) an external researcher was employed to conduct the interviews and the initial coding, c) there were joint discussions between three researchers with different perspectives based on education and experience, and d) the authors (CW) who provided almost all the information sessions did not take part in the thematic analysis.

## Results

### Study participants

**Fig. 1:**
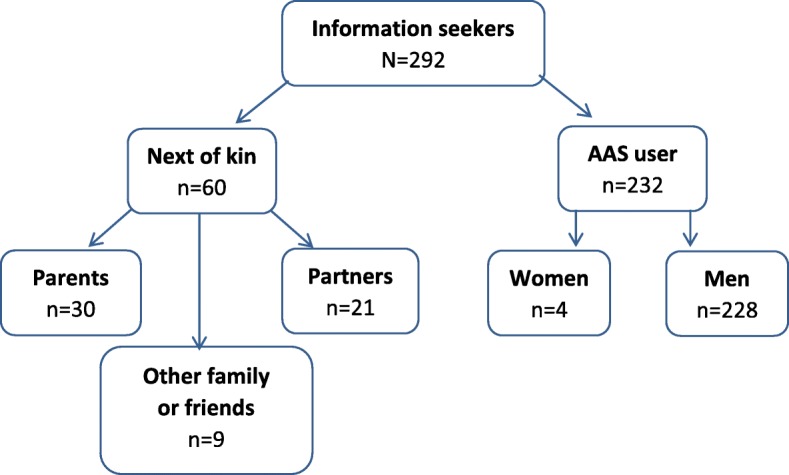
Individuals (*n* = 292) who contacted the Steroid Project for an information session regarding possible health problems related to AAS use and treatment possibilities

Over a period of four years, 292 individuals from all four health regions in Norway contacted the national Steroid project at Oslo University Hospital for an information session. 232 (79.5%) had experience with use of AAS and 60 (20.5%) were next of kin of AAS users. There were 228 (98.3%) men and 4 (1.7%) women who reported using AAS (Fig. 1). 37 individuals used the service in 2015, 40 in 2016, 45 in 2017, and from January through March 2018 only 8 used the service. But after the social media campaign started in April 2018 through March 2019, 162 individuals used the service.

### Information sessions

One hundred and eight (37.0%) information sessions were conducted as personal meetings, 182 (62.3%) as telephone meetings, and two (0.7%) by mail. 53.8% of the information seekers became acquainted with the service via Internet/media. The remaining got information from the following sources: Antidoping Norway (11.3%), health staff (13.4%), family/friends (12.0%), Police/Prison authorities (2.7%), and other/missing (6.5%).

### Sample descriptions AAS users

The mean age of the 232 AAS users was 31.4 years (median 28, range 16–67). The mean age for first time AAS use was 21.2 years (19, 12–51) and average length of use was 10.2 years (8, 0.1–40). The majority of the AAS users were either employed (*n* = 118, 50.9%) or students (62, 26.7%). 11 (4.7%) were on sick leave and 25 (10.8%) received unemployment or disability benefits, 8 (3.5%) were unemployed, and eight (3.5%) did not give their employment status. 107 (46.1%) had a partner, 88 (37.9%) did not, and 37 (15.9%) gave no information regarding their marital status. 75 (32.3%) of the users had children, 114 (49.1%) did not, and 43(18.6%) did not provide information about parental status. 110 (47.4%) of the AAS users informed that they used or had used one or more drugs and addictive substances in addition to AAS.

### Physical and mental health problems

**Fig. 2:**
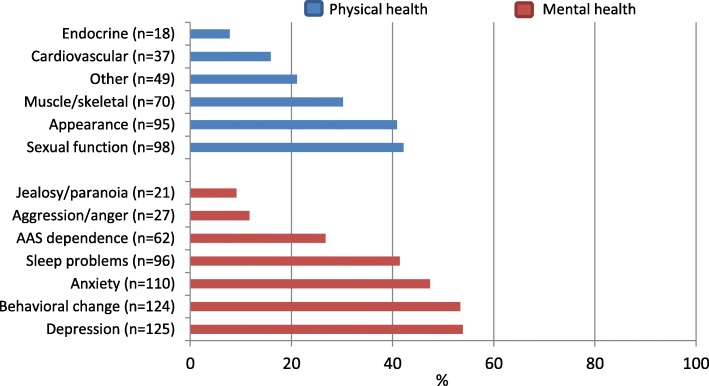
Reported physical and mental health problems related to use of AAS (*n* = 232), proportions (%). Multiple answers were possible

One hundred and eighty-one (78.0%) of the 228 male and four female AAS users reported physical and mental health problems related to AAS use, 31 (13.4%) had only experienced mental health problems, 16 (6.9%) had only experienced physical, and four (1.7%) had not experienced any side effects related to AAS use. Depression, changed behavior and anxiety were the most common mental conditions reported. The most frequently reported physical health problems were related to sexual function, appearance and muscle/skeletal conditions (Fig. 2).

The 110 AAS users who reported illicit substance use had a significantly higher mean number of mental health problems (M = 2.8, SD = 1.3) when compared with the 28 who reported no substance use (2.3, 1.2); t (136) = 2.20, *p* = 0.029, 94 were missing.

All four women included in Fig. 2 reported depression, anxiety and/or behavioral change, and two or more of the following masculinizing side effects: facial hair, body hair, deepened voice, reduced breast volume, a more masculine look and loss of menstrual cycle. In addition, physical health problems such as acne, sweating and cardiovascular disease were reported.

### Reported observations by next of kin

**Fig. 3:**
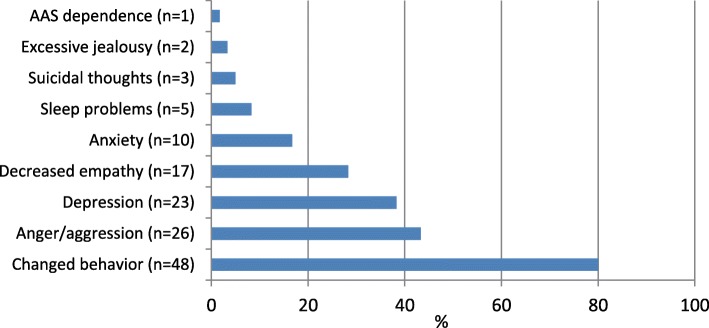
Observed changes of the AAS user, as described by next of kin (*n* = 60), proportions (%). Multiple answers were possible

Fifty-eight of the next of kin had registered that the AAS user had changed mentally, and most commonly reported changed behavior (*n* = 48, 80.0%), anger or aggression (26, 43.3%), depression (23, 38.3%), decreased empathy (17, 28.3%) and anxiety (10, 16.7%) (Fig. 3).

### Motivation for AAS cessation and treatment desire

Of the 232 AAS users, 226 were motivated to or had already stopped using AAS, and contacted the information service because they were interested to know what help they could be offered from the health care system. The other six were not motivated to stop using AAS, but took contact because they wanted harm reduction advice regarding their AAS use. The most common motivation for AAS cessation was mental health problems (*n* = 108, 47.8%), followed by a combination of mental and physical health problems (52, 23%), physical health problems (34, 15%), other motivations (27, 11.9%) and missing (5, 2.2%),

Of the 232 AAS users, 179 (77.2%) reported that they desired treatment after completing the session, 32 (13.8%) were unsure, and 21 (9.1%) did not want treatment. Among those who desired treatment, 158 (88.3%) wanted referral to SUD treatment, and 21 (11.7%) were motivated to get other treatment by contacting their general practitioner directly. The AAS users who desired treatment were older, had used AAS longer, had a higher proportion with children and also reported a higher mean number of physical, mental and total health problems than those who were unsure or did not want treatment (Table 1).

Six (10%) of the 60 information-seeking next of kin wanted treatment after the information session, nine (15%) were unsure, 36 (60%) did not want treatment, and nine (15%) we lack information on.

### Perceived service satisfaction

Of the 232 AAS users, 228 (98.3%) considered the information consultation as useful in the sense that the information gained was important to be able to decide whether they wanted SUD treatment in the specialist health service or not, three (1.3%) reported that it was not useful, and one (0.4%) did not respond. All 60 (100%) next of kin considered the information session important to understand side effects of AAS use and where to seek treatment for the AAS user or oneself.

### Questions asked by the AAS users

Frequently asked questions from the AAS users prior to deciding whether they wanted help were whether health personnel were obliged to report to the police, child welfare, employer, traffic authorities or others. In addition, they wanted information about conditions for receiving testosterone replacement therapy, what examination and treatment options could consist of and what possible treatment outcome could be expected, especially in relation to mental health symptoms. The themes brought up were later covered in the information sessions.

### The information service may reduce the barriers to seeking treatment

This section will focus on understandings and experiences of the information service and data are drawn from a qualitative study exploring barriers to treatment and treatment experiences. Five of the study participants had used the service prior to entering outpatient SUD treatment. Further two had positive treatment experience and got information about the service after they had entered treatment and advised friends and acquaintances with AAS-related health problems to use the information service. Findings from these interviews suggest that the information service may reduce the barriers to seeking treatment through:

- organizational factors, as the service is organized to be flexible and easily accessible;
- informational factors, including information about what addiction treatment actually is and the ability to tailor the information to meet the needs and fears of the service user;
- facilitating the treatment entry process – and establish contact with health professionals who have experience with treatment of individuals with AAS-related health problems; and
- individual factors of the service provider, including personal engagement, knowledge about AAS use, side effects and addiction treatment.

Here we present some cases. A 30-year-old man had used AAS for a year during his early twenties, he then tried to quit and restarted use due to severe depression and fatigue. After that, he used AAS almost continuously for several years and asked his GP to help him but was always told that he had to stop using AAS and wait until the endogenous testosterone production improved. He described how getting access to the information service was central in managing to quit AAS:

*"The second period I didn’t want to be on steroids. It was always my intention not to be on steroids. I have tried to quit many, many, many times, but never got the help I needed to do it. I contacted my GP many times without getting help. So yes, I wanted to stop many times. [..] I was lucky because last time I went to the GP, it was a new physician and she had a flyer about the Steroid Project [providing the information session]. She was completely honest and said: “I know nothing about it [treatment] and I know nothing about steroids, but they seem to have some knowledge. It says that they have a treatment system and that it is anonymous”. Then I said: “Just do whatever is needed. I just want to quit”.*

*The GP called the Steroid project and booked an appointment a few hours later and during the information session he remembered that he said:*

“*… if you help me NOW, I will give you my bag of steroids. And they did. They took the bag and called the addiction outpatient department right away. I got my first appointment the day after”.*

When asked how he experienced the information session he responded:

*“It was very good. It wasn’t negative or judgmental at all. [..] … it was very nice to feel that somebody actually cared about my desire to quit and they didn’t act cool or want help [from me], but sent me to the right person right away. And I met the right psychologist, he was the one who made me stay away. Regular appointments every other day or weekly”.*

To him, it was essential that health staff providing the information service was easily accessible, flexible, knowledgeable and cared about his wish to quit, and showed it by guiding him into treatment shortly after the information session, with health professionals who had experience with treatment of AAS-related health problems.

A 40-year-old participant had used AAS for about 10 years and experienced various mental and physical side effects. He had only sought help for these side effects in the «AAS-using community» and explained that he then did not contact the health services because he believed that the level of knowledge among health professionals was low. He also had substance problems and finally managed to end both illicit substance and AAS use with support from health professionals at an outpatient addiction treatment. He did not use the information service prior to entering treatment, but got acquainted with it later and advised some of his AAS-using friends to use the service. He clearly states that the information service should be a part of the specialist health service and at the same time must be a low-threshold and easily accessible service in order to be used, and that the main challenge is to provide information about what addiction treatment actually is – so that AAS users with health problems who are skeptical towards health services may choose to enter treatment:

*“It is important that a low threshold service, such as the Steroid project, has no need for referral from a GP. You can just call or send a text message. I think that is very important [..] – Still, overcoming the threshold to become a patient, getting a medical file and all that – makes it quite a task to inform [AAS-users] that it [being in outpatient addiction treatment] is no harder than talking to a pal. It [the information service], I think, needs to be a part of the treatment system to make treatment more accessible for more [AAS-users]. To receive an appointment for a meeting and just arrive in an office, or a medics office or something like that, that setting may be enough to avoid seeking help [in the specialist health service]”.*

Another participant had used the service while he was imprisoned and pointed out that being able to access to the information service without a referral, as the referral process is time-consuming for prisoners, and having a noncommittal meeting were important prior to deciding if he wanted treatment. The service provider could also help by speeding up the referral process for treatment:

*“Yes, I think it is likely to be decisive if you can have a noncommittal meeting without a referral, particularly for people like me who are imprisoned, because in prison everything is so difficult. There is so much waiting, including for referrals. But with an information session you may have one [referral for AAS addiction treatment] after a week or so, that is good”.*

A man in his forties had entered addiction treatment after near two decades of AAS use; the last 10 years were characterized by continuous use of high doses in combination with illicit substances. After having positive treatment experiences and outcomes he recruited several AAS users both to the information service and outpatient addiction treatment. He reflects on the fact that AAS use and possession is illegal in Norway and that AAS users may fear negative sanctions, particularly for those with co-occurring substance use disorders. Therefore, he thinks that a noncommittal and anonymous information session focusing on realistic reporting practices in addiction treatment is of great importance, including assurance that AAS users will not be reported to the police, when deciding if they will seek help in the health services to end their AAS use:

*“I think it [the information service] is very good, to put it like that. People are afraid to lose their lives, lose their children, lose their driver’s license, lose their job – who would want that? [..] … and the informal conversation you can call for and inquire about and bla bla bla. Then you might prepare a bit: OK, I might give up my driver’s license for half a year, OK. Child protection might come into play – OK, but everything is fine at home, I haven’t done anything wrong. They will not take my kids. I am in treatment now, I’m getting help, and the kids are fine with their mother, right. So I think it has to do with taking away the fear”.*

## Discussion

Information sessions with health professionals that covered treatment options were utilized by 232 AAS users and 60 next of kin over a four-year period from 2015 to 2019. 179 (77.2%) of the 232 AAS users desired treatment after receiving the information. Those who desired treatment were older, had used AAS longer, reported more side effects, and a higher proportion reported having children than those who did not desire treatment. Although 181 (78.0%) of the AAS users reported both mental and physical health problems related to AAS use, mental health problems (*n* = 108, 47.8%) were the most common motivation for ending AAS use, followed by a combination of mental and physical health problems (52, 23.0%),

### Use of health-related information service may facilitate treatment seeking

AAS users may get information about AAS and health related issues from different sources; with various motives of providing the information; online bodybuilding stores [52], individual sellers of AAS and other doping agents [53], individuals in the gym environment and online forums where the identity of the forum user is unknown [54, 55]. A health staff driven information service where the information seeker can be anonymous, may be a neutral information source for AAS users with health problems and their next of kin. It was central to the information seekers that they could ask questions about treatment options and possible treatment outcomes. Furthermore, prior to deciding whether they desired SUD or other treatment, the AAS users needed to know whether health personnel were obliged to report to the police, child welfare, employer, traffic authorities or others. It is therefore likely that the Norwegian legislation in 2013 that made AAS use and possession illegal, may act as a barrier to contacting health services for AAS users.

Few AAS users are found to seek medical assistance for side effects related to AAS use [44, 56, 57], but a recent study found that AAS users had more often hospital contacts than non-using controls [30] and another study among current AAS users with health concerns, only 35% reported engagement with health services during the last year [44]. Still, there are AAS users who do not feel they need health services as they experience few and less serious side effects and manage to end their AAS use without external support [58] and some AAS users do not find their conditions to be significant enough, nor do they believe that physicians can or will help [44]. However, those in need of health services wish to be met without judgements, and by health professionals with knowledge about AAS [58, 59], and they were positive towards getting a health evaluation [53, 58]. It is found that health professionals may possess negative attitudes and may stigmatize AAS users [60]. Additionally, some AAS users perceive health professionals as unknowledgeable about AAS [44, 61]. These factors may be of importance when trying to understand why many AAS users avoid seeking health care. An information session where the information seeker can discuss health issues with non-judgmental health professionals that are experts on health problems related to AAS use may therefore be experienced as a positive “first step” and may facilitate treatment-seeking for AAS users in need of health services.

### Health problems and behavioral change among AAS users

Concurrent mental and physical health problems were reported by 78.0% of the AAS users, and those who desired treatment reported a higher mean number of side effects than those who did not desire treatment. Sexual problems and health problems regarding image and muscle/skeletal problems were the most commonly reported physical health problems. Sexual dysfunction is found to be common among AAS users, particularly after ending AAS use [16, 17] and it has been found that sexual problems increased the likelihood for contacting a physician [44]. Endocrine disturbances were reported by 7.8% and cardiovascular conditions were reported by 15.9% of the AAS using information seekers in our study and some of the conditions were serious. It is important to note that several medical conditions can be present without symptoms and instead must be diagnosed by medical examinations and more participants than reported may therefore have pathological findings. However, the most common motivation for the desire to discontinue use of AAS was mental health problems. Depression and anxiety were the most frequently reported mental health problems, and 26.7% of the AAS users in our sample stated that they experienced dependency to AAS. Individuals reporting AAS dependence may carry a higher risk of physical and mental health problems as AAS is taken in higher doses, with shorter breaks or continuously despite such side effects, and SUD treatment should therefore be available for those in need [22, 23]. Mood swings during AAS use is frequently reported as well as symptoms of depression and anxiety, particularly during periods of abstinence [2]. As AAS use may be a risk factor for suicidality [62–64], an important measure will be to conduct suicide risk assessment and offer treatment of depressive conditions among AAS users [22]. Of note, it has been reported that AAS users who had contacted a physician for health problems were pleased with the healthcare services when mental health problems were addressed as a topic [44].

Furthermore, 47.4% of the users of AAS reported having used illicit drugs and/or addictive substances together with AAS, as is commonly reported by AAS users [38–42, 45], 46.1% stated that they had a partner, and 32.3% reported having children. Among the 60 next of kin who received information sessions, it was common to describe the individual using AAS as exhibiting changed behavior, anger/aggression and depression. Although their life situation was described as demanding and stressful, only six wanted treatment for their own sake. Changed behavior, mental health problems and substance use among some AAS using individuals may affect parents, partners and children. Our findings suggest that social issues, aggression and substance use should get attention and be a part of a treatment program when present, and there is a need to explore what kind of services next of kin are motivated to receive or take part in.

### Strengths and limitations

This study provides new and clinically relevant information based on information sessions conducted as personal meetings with a sample of AAS users and next of kin where almost all the users described AAS-related health problems and a wish to end AAS use permanently and the vast majority desired treatment to reach their goals. Next of kin to AAS users are included in the study as they provide an important perspective. However, this study has several limitations, as it was conducted to document and improve the information service for AAS users and next of kin. The occurrence of health problems may show lower incidence, and some illnesses may have occurred before the user started with AAS as *all* possible adverse reactions, health status prior to AAS use and all previous or current illicit substance use were not systematically questioned in this survey. Furthermore, the participants were not tested for AAS dependence and there may therefore be a higher proportion of AAS users with AAS dependence than reported. Years of AAS use may not be an optimal measure of severity of use as the study does not distinguish between cyclic or continuous use, doses and specific substances. It was common that the participants had tried to end their AAS use several times without succeeding, some had just restarted and others had reduced their dose or ended their use just prior to the information session. Therefore, it was problematic to distinguish between previous and present use. This article deals with a self-selected group of AAS users with mental and/or physical health problems who have contacted the information service. Those who voluntarily seek information about treatment of adverse effects related to AAS use are probably already motivated for entering SUD treatment or other treatment. Hence the desire for treatment is higher among the information seekers than for all AAS users with health problems. Although three out of four of the information seeking AAS users desired treatment, data on the proportion who actually entered treatment was not collected as the project did not have ethical permission to do so. Norway has a national patient registry (NPR) and future research should focus on whether more patients entered the specialist health services during the intervention period by use of NPR or other data sources. Qualitative data from participants in a study about barriers to seeking treatment were included in this paper to provide information about how the service may be of use to overcome treatment barriers. We have not included participants who did not want to cease AAS use or did not desire addiction treatment after the information session. Furthermore, the next of kin may have a relationship with a subgroup of AAS users with mental and behavioral changes in relations to AAS use. Findings in this article can therefore not be generalized to all users of AAS with or without health problems and their next of kin.

## Conclusions and clinical implications

The information service was used by a substantial amount of individuals with AAS-related health problems who struggled to end their AAS use. It is important to make such a service known and available for potential users and this study suggests that an information service may reduce barriers to seeking SUD or other treatment. It is worth noting that many users have skepticism towards all health services and need reassurance against negative sanctions and dissemination of their difficulties. Furthermore, the dialogue based information sessions reveals extensive physical and mental health problems among the information seekers and the user group is so complex and non-homogeneous that examination and treatment must be adapted individually with focus on physical, mental and social factors as well as possible addiction of AAS and/or psychoactive substances. Healthcare professionals who encounter potential users of AAS needs to be non-judgmental and should have knowledge about AAS use and possible side effects to provide relevant information and initiate physical and mental health examinations, map substance use if present, and social conditions so that personalized health care can be offered.

## Abbreviations

AAS: Anabolic androgenic steroids
SUD: Substance use disorder
